# King Edward's Hospital Fund for London Statistical Report

**Published:** 1920-01-31

**Authors:** R. Orde

**Affiliations:** House Governor and Secretary. Royal Victoria Infirmary, Queen Victoria Road, Newcastle-on-Tyne


### THE EDITOR'S LETTER-BOX.

### KING EDWARD'S HOSPITAL FUND FOR LONDON STATISTICAL REPORT.

### The Figure Cost per Occupied Bed.

*To the Editor of* THE HOSPITAL.

SIR,—I am glad to see in your issue of January 10 that you question the value of the figure cost per occupied bed, except, of course, for home consumption. It is difficult to see how this figure can be accepted as a criterion of efficiency or economy. One hospital with a low cost may be inefficient, spending its money to little purpose; while another with a high cost may be efficient and may get full value for the money it spends. Two hospitals, one with a high cost, the other with a low cost, may, both of them, be efficient and economical. One body of managers may take the view that a hospital fulfils its function if it treats the disease; another that, in addition to treating the disease, the cause must be studied and the means of prevention discovered. Is the second to be condemned because it spends more than the first? Is the first to have the advantage of all the discoveries made by the second and be subsidised into the bargain because of its low cost? Further, the figures in the statistical tables which go to make up the cost per occupied bed cannot be considered comparable. How, for example, can two hospitals be compared in the matter of their domestic expenditure when water may be a free gift to the one and cost anything up to £1,000 a year to the other? (There are, of course, numberless other instances of similar discrepancies.)

Thus, as you rightly point out, before coming to any conclusion it is necessary to know more than the statistical reports are able to tell. If grants are to be made to hospitals on the basis of efficiency and economy then some other criterion than is furnished by any set of hospital figures as at present drawn up is earnestly to be desired.

I would suggest that it is not only possible but easy to draw up a series of questions, the answers to which would furnish a good working basis for any distributing committee, provided that these answers are supplemented, wherever necessary, by a personal visit to the hospital. Provided, further, that such a visit is made by two or three men intimately acquainted with hospital work; that they spend adequate time on their inquiry; that the hospital authorities have ample opportunity of putting their visitors in full possession of all the facts; that any report which the visitors draw up be first submitted to the hospital authorities for any comments they may be able to make. Hospitals differ so much, and there are so many qualifying factors, that unless this is done a hospital may be most unfairly penalised solely because its cost per occupied bed is high.—I am, yours, etc., R. ORDE, House Governor and Secretary.

Royal Victoria Infirmary, Queen Victoria Road, Newcastle-on-Tyne, January 20, 1920.

